# THE SPILLOVER EFFECTS OF COMPREHENSIVE CARE FOR JOINT REPLACEMENT (CJR) MODEL: A STUDY FROM CALIFORNIA

**DOI:** 10.1093/geroni/igad104.1992

**Published:** 2023-12-21

**Authors:** Narae Kim, Mireille Jacobson

**Affiliations:** University of Southern California, Los Angeles, California, United States; University of Southern California, Los Angeles, California, United States

## Abstract

In 2016, Medicare implemented the Comprehensive Care for Joint Replacement (CJR) program to test whether paying hospitals a bundled payment for 90 days of an episode of care for lower extremity joint replacement (LEJR), the most common surgery for Medicare beneficiaries, can improve care coordination and quality in traditional Medicare (TM). Unlike most Medicare alternative payment models, CJR participation was randomly assigned across Metropolitan Statistical Areas (MSAs). Hospitals in selected MSAs were required to participate in the program, while hospitals in other MSAs were paid in the usual fee-for-service manner. Taking advantage of this random assignment, we examined CJR’s direct effects on TM patients and spillover effects on Medicare Advantage (MA) and non-Medicare patients in California. Using California’s Patient Discharge Data (PDD) from 2014 to 2017and event study and difference-in-differences models, we studied changes in adjusted length of stay and home discharge rates before and after program implementation in hospitals in treated versus control MSAs. We found that the CJR program affected not only TM patients, but also untargeted MA and non-Medicare patients. Both TM and non-Medicare patients in treated hospitals experienced shortened length of stay (-3.9% & -1.3%, p<0.05) and increased likelihood of discharge home (3.4%, 2.3%, p< 0.001) relative to those in untreated hospitals after program implementation. MA patients experienced an increase in not only home discharge rates (4.7%, p< 0.001) but also length of stay (2.5%, p< 0.01). Programs designed to affect Medicare costs have the potential to affect the care of patients not covered by the program.

